# Study on Mechanical and Electrical Properties of High Content CNTs/Cu Composites

**DOI:** 10.3390/ma17153866

**Published:** 2024-08-05

**Authors:** Ziyang Xiu, Jinpeng Sun, Xiao Li, Yihao Chen, Yue Yan, Puzhen Shao, Haozhe Li, Boyu Ju, Wenshu Yang, Guoqin Chen

**Affiliations:** 1 State Key Laboratory of Precision Welding & Joining of Materials and Structures, Harbin Institute of Technology, Harbin 150001, Chinayws001003@163.com (W.Y.); 2 Beijing Institute of Space Mechanics & Electricity, Beijing 100941, China; 3 Feiteng Technology (Changsha) Co., Ltd., Changsha 410003, China; 4 Zhejiang Academy of Special Equipment Science, Hangzhou 310020, China; 5 Zhengzhou Research Institute, Harbin Institute of Technology, Zhengzhou 450046, China

**Keywords:** CNTs, Cu matrix composite, sintering, directional rolling, properties

## Abstract

It is expected that composites made of carbon nanotubes (CNT) and copper (Cu) display both mechanical and electrical properties, but the low damage dispersion and high-quality composite of high-content CNTs have always been research difficulties. In this paper, high-content CNTs/Cu composites were prepared. The effects of the sintering method, sintering temperature, directional rolling and the CNTs’ content on the relative density, hardness and electrical conductivity of the composites were studied. The uniform dispersion of high-content CNTs in Cu matrix was achieved by ball milling, sintering and rolling, and the processes did not cause more damage to the CNTs. The properties of composites prepared by spark plasma sintering (SPS) and vacuum hot pressing sintering (HPS) were compared, and the optimum process parameters of SPS were determined. When the CNTs’ content is 2 wt.%, the hardness is 134.9 HBW, which is still 2.3 times that of pure Cu, and the conductivity is the highest, reaching 78.4%IACS. This study provides an important reference for the high-quality preparation and performance evaluation of high-content CNTs/Cu composites.

## 1. Introduction

With the development of electrical and electronics, transportation, aerospace and other industries, the requirements for the strength and conductivity of materials are constantly increasing [1,2,3,4]. It is necessary to continuously develop new lightweight materials with sufficient strength [5], electrical conductivity [6,7] and thermal conductivity [8]. CNTs have unique structures and excellent theoretical properties, with extremely high Young’s modulus, tensile strength and electrical conductivity [9]. As a reinforcement, CNTs have been widely used in Mg-matrix [10], Al-matrix [11,12], Cu-matrix [13], Ti-matrix [14] and other metal systems.

A large number of studies have shown that the addition of CNTs can improve the mechanical and electrical properties of Cu [15,16,17]. Shukla [18] prepared copper based composites reinforced with single-walled carbon nanotubes (SWCNTs) using high-energy ball milling and vacuum hot pressing methods. With the increase of CNT content, the hardness of the composite significantly improved. Qiang Lu’s [19] studies have shown that the addition of a low volume fraction of CNTs (0.5 vol.%) can improve the friction resistance of the composites under dry reciprocating sliding conditions. The conductivity increases slightly with the addition of appropriate content of CNTs, and the composite with 1 vol.% CNTs shows the best conductivity. Shaoli Fu et al. [20] successfully prepared Cu/CNTs composites by two different precursor preparation techniques: the alloying method and the co-deposition method, chemical vapor deposition and spark plasma sintering. The hardness reaches 102.5 HV, which is 24.7% higher than that of pure copper, and the tensile strength is 275 MPa, which is 36.1% higher than that of pure copper. In addition, the conductivity remained at a high level of 92.9% IACS. The addition of CNTs can improve the mechanical, electrical and thermal properties of the composites, and it is expected to prepare composites with high strength and high conductivity [2,21]. However, some studies [22,23] have shown that, when the content of CNTs is too high, the addition of CNTs not only reduces the conductivity of CNTs/Cu composites, but also adversely affects their mechanical properties.

In order to improve the final performance of the composite, the preparation process needs to be improved. Xinhua Wang [24] found that the interfacial bonding of Cu/CNTs composites is highly dependent on the sintering temperature and CNTs’ content. The relative density of the prepared composites increases with the increase of sintering temperature. Increasing the sintering temperature can enhance the diffusion of atoms, which helps to eliminate the pores in the Cu matrix. A high sintering temperature can promote the infiltration of Cu atoms into the inner wall of CNTs through defects on the CNTs wall, thereby enhancing the interfacial bonding of Cu-CNTs. Siwei Luo’s research [25] found that coating Cu on CNTs can not only promote the dispersion of CNTs and enhance the CNT-Cu interface, but also make the strain distribution of the composite uniform and form a dislocation elimination zone in the interface area, so as to obtain a better strength-plasticity synergistic effect than uncoated CNTs reinforced Cu matrix composites. Liu [22] successfully prepared uniformly dispersed CNTs/Cu composite powder by spray pyrolysis, and prepared CNTs/Cu composite by SPS sintering technology. The material has high relative density, the ultimate tensile strength is 344 MPa and the elongation is 19.6%. However, the content of CNTs in the previous studies was relatively small, and the properties of the composites did not reach the ideal level. In order to obtain better mechanical properties, we studied how to prepare composites with higher CNTs content.

In this study, we used the wet high-energy ball milling method to disperse CNTs, compared the two sintering methods of SPS and HPS, and selected the SPS method with higher relative density and better interface bonding. The effects of sintering temperature and directional rolling process on the mechanical and electrical properties of graphene were studied. The relative density, hardness and conductivity of the composites were characterized. Finally, the optimum process parameters were determined, which provided guidance for the preparation of high content CNTs/Cu composites.

## 2. Experimental Investigation

### 2.1. Preparation of CNTs/Cu Composites

**Figure 1:**
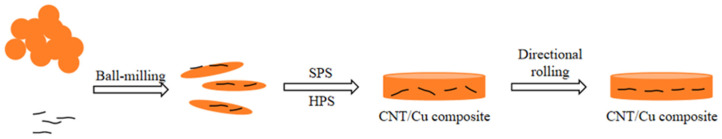
Preparation process of CNTs/Cu composites.

The matrix used in this experiment is nano copper powder. Nano copper powder is produced by Lebo Metal Materials Technology Co., Ltd. (Xingtai, Hebei, China). The diameter of nano copper powder is 600 nm, and the composition is 99.9 wt.% copper. The reinforcement used in this experiment is CNTs produced by Shenghui New Carbon Materials (Nanyang, Henan, China). The purity of the CNTs is more than 95%, the inner diameter is 3–5 nm and the length of the tube is 3–12 μm. It has a high aspect ratio and intertwined surface morphology. Raman characterization I_D_/I_G_ is 1.13, the degree of damage is low and the structural integrity meets the experimental requirements. CNTs/Cu composites were prepared by SPS and HPS. The main preparation process is divided into three stages: high energy ball milling, sintering and directional rolling optimization, as shown in Figure 1. The ball milling process is: speed 200 rpm, ball-to-powder ratio 10:1, ball milling time 8 h. The directional rolling parameters are as follows: rolling temperature 800 °C, single rolling amount 5% and holding time between two adjacent rolling for 10 min.

### 2.2. Material Characterization

Density testing (the density measuring equipment model is LABOX-325R, from METTLER TOLEDO Technology Co., Ltd., Shanghai, China) using a drainage method to test the relative density of composites, using deionized water (density ~1 g/cm^−3^) as the testing medium. The relative density is obtained by dividing the measured actual density by the theoretical density. The density used in the theoretical density calculation: the density of copper is 8.96 g/cm^3^, and the density of CNTs is 1.8 g/cm^3^.

Brinell hardness test (the hardness measuring equipment model is HBV-30A, from Hangzhou Laihua Test Instrument Co., Ltd., Hangzhou, Zhejiang, China) using a Brinell hardness tester to test the hardness of composites, unit HBW. The quenched steel ball is pressed into the surface of the tested metal material with a certain amount of load, and the load is removed after holding for a period of time. The hardness was calculated by measuring the diameter of the steel ball pressed into the microscope.

Conductivity test (conductivity measurement equipment model is FD-102, from Foster Electronic Technology Co., Ltd., Xiamen, Fujian, China) using the FD-102 eddy current conductivity meter manufactured by FIRST company to test the conductivity of composites, at a test temperature of 25 °C. % IACS is the unit of conductivity, which represents the percentage of the ratio of the conductivity of the sample to a certain standard value. The sample size of the conductivity test is 10 × 10 × 1 mm.

The characterization of the microstructure of composites using scanning electron microscopy (the equipment model is FEI Quanta 200FEG, from Shanghai, China). Characterizing the surface of composites using a metallographic microscope (the equipment model is Axiovert 40 MAT, from Zeiss, Oberkochen, Germany) to observe whether there are obvious pores and cracks in the composite. 

Raman spectroscopy (the equipment model is Zolix Omni λ500i, from Beijing Zhuoli Hanguang Instrument Co., Ltd., Beijing, China) is used to characterize the lattice integrity of CNTs. Select a 600 aperture, test laser wavelength of 532 nm, and test range of 500–3000 cm^−1^. The degree of damage to CNTs during ball milling can be characterized by the peak intensity ratio (I_D_/I_G_) of the D and G peaks in Raman spectroscopy. The higher the ratio, the higher the degree of defect in CNTs.

## 3. Results and Discussion

### 3.1. The Mode of Sintering

**Figure 2:**
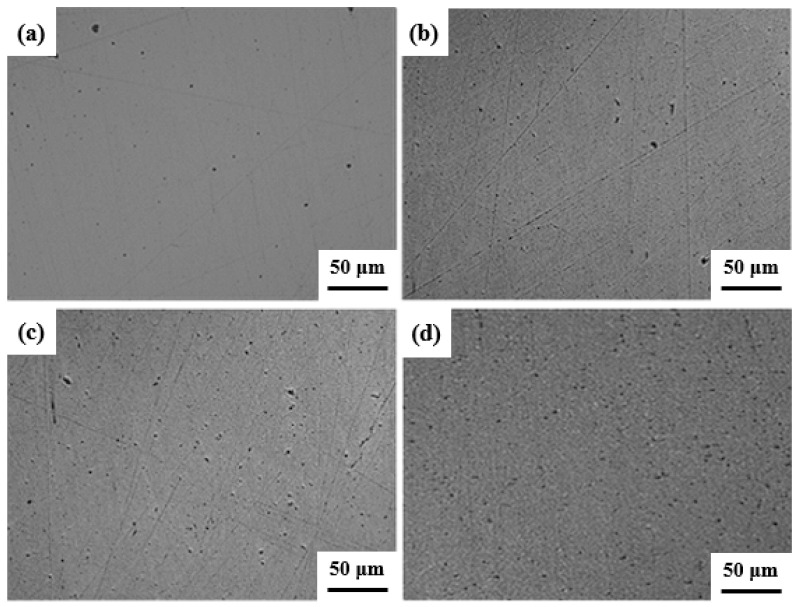
Microstructure of CNTs/Cu composites under different sintering methods. (**a**) HPS, 1 wt.%CNTs; (**b**) HPS, 2 wt.%CNTs; (**c**) SPS, 1 wt.%CNTs; (**d**) SPS, 2 wt.%CNTs.

In order to study the effect of the sintering method on the relative density of composites, composites with process numbers 1-1 to 1-4 were prepared, and the microstructure photographed by SEM is shown in Figure 2. Compared with the same CNTs content, the surface pores of CNTs/Cu composites sintered by HPS are obviously more than those sintered by SPS. Although the size of CNTs is small and difficult to observe through metallographic microscopy, the dispersion and material relative density of CNTs can be indirectly judged by observing the pores generated by CNT aggregation.

The density, relative density, hardness and electrical conductivity of the composites prepared by different processes are shown in Table 1. The source of the error is the experimental error, because the composite material is not completely uniform, and each data is measured at least three times. Comparing the two sintering methods, the relative density of CNTs/Cu composites prepared by SPS is generally higher than that of HPS. The highest relative density of the composites prepared by the SPS method reached 92.0%, while the highest relative density of composites prepared by HPS was only 86.1%, with a difference of 5.9%. The relative density of CNTs/Cu composites prepared by SPS is higher, which is caused by the characteristics of SPS sintering itself. Although SPS and HPS are sintered by heating and pressing, the heating methods of the two are different. HPS is heated up by heating the resistance to produce radiation, while SPS is heated under the action of pulse current. The nano Cu powder used in the experiment has a strong adsorption capacity due to its large specific surface area, which makes it difficult to discharge some gases during the sintering process. The discharge effect of SPS sintering can clean up the gas adsorbed on the surface and inside of the powder. In addition, the SPS sintering time is short, and the agglomeration of CNTs in a short time is less, which greatly reduces the porosity of the material, thus making the material more dense. When the CNTs content is the same, the hardness and conductivity of the CNTs/Cu composites prepared by SPS method are higher than those of HPS, which shows the same law as the relative density of the material.

Therefore, by comparing the changes of microstructure, relative density, hardness and conductivity of the composites prepared by the two processes, SPS was selected as the final sintering method, and the sintering temperature was further optimized to further improve the relative density of the composites. 

### 3.2. Sintering Temperature

In the process of preparing CNTs/Cu composites by SPS, the sintering temperature is the parameter that has the greatest influence on the relative density of the composites. SPS belongs to low temperature sintering, and the sintering temperature is generally 60~90% of the melting point of the material. The melting point of pure Cu is 1084 °C, so the sintering temperature is set to 600 °C, 700 °C, 800 °C, 900 °C and 1000 °C, the heating rate is 120 °C/min, and the holding time is 20 min.

**Figure 3:**
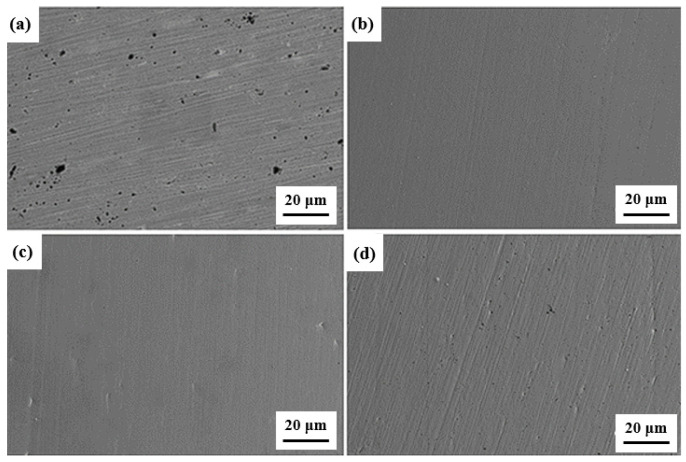
Surface morphology of composites at different sintering temperatures. (**a**) 600 °C; (**b**) 700 °C; (**c**) 800 °C; (**d**) 900 °C.

Figure 3 shows the surface morphology of the composites at different sintering temperatures, taken by SEM. At 600 °C, the relative density of the composites is much lower than that of the composites prepared at other temperatures. The microstructure is shown in Figure 3a. It can be seen that there are a large number of pores on the surface of the composites, and the distribution of pores is not uniform. This is because at a lower temperature, the diffusion coefficient between the components of the composite is small, the composition is difficult to homogenize in a short time and the interface between the Cu matrix and the CNTs is not close, resulting in a large number of pores, an uneven material composition and a low relative density. At 700 °C, the surface of the material is relatively smooth, and no holes are basically observed. At this time, the material has a high degree of densification. At this time, the composite may show ideal performance, as shown in Figure 3b. With the further increase of temperature, the existence of pores can be observed on the surface of the material when the sintering temperature reaches 800 °C. This is because the agglomeration of CNTs is pinned at the grain boundary of the material, which makes it difficult to further densify the material. When the sintering temperature reaches 900 °C, the number of pores on the surface of the composite increases further, which will undoubtedly lead to a decrease in the relative density of the material, thus adversely affecting the poor performance of the material. At 1000 °C, the SPS sintering temperature is too high, resulting in a large amount of liquid phase, which leads to the melting of the composite and the outflow of the graphite mold. In the sintering process, the sintering temperature should be controlled at 900 °C and below.

**Figure 4:**
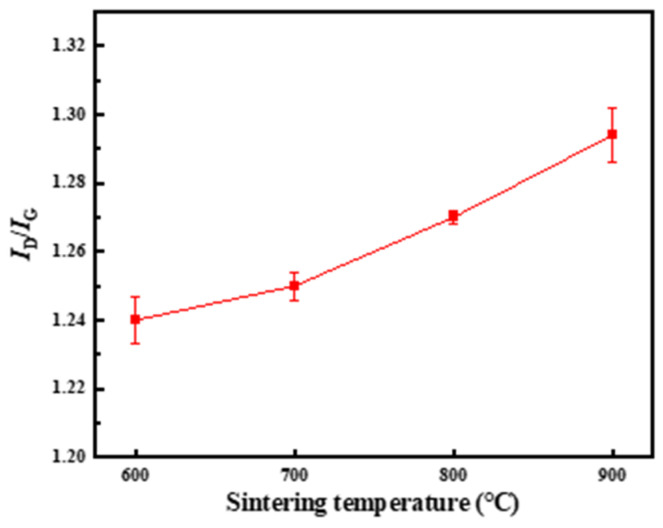
The effect of sintering temperature on the I_D_/I_G_ of CNT.

It can be seen from Figure 4 that as the sintering temperature increases, the value of I_D_/I_G_ gradually increases, indicating that the defects of CNTs gradually increase during the sintering process. This is due to the diffusion of C atoms in CNTs at high temperature, which destroys the crystal structure of CNTs and increases the content of defects. In general, between 600 °C and 700 °C, the value of I_D_/I_G_ rises slowly, and the damage caused by sintering to CNTs is small. When the temperature continues to rise, the value of I_D_/I_G_ rises rapidly. Combined with the microstructure of the composite, it can be considered that this is due to the secondary agglomeration of CNTs, which leads to the destruction of the CNTs’ structural integrity.

**Figure 5:**
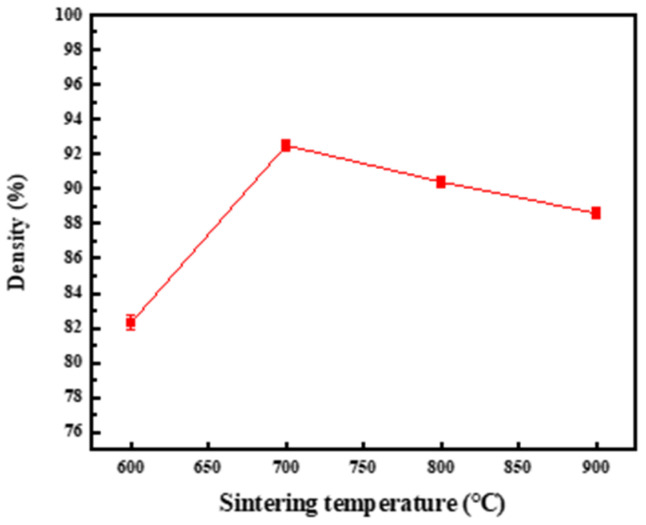
Variation of relative density of composites with sintering temperature.

Table 2 shows the relative density and relative density of 1.5 wt.% CNTs/Cu composites at different temperatures measured by the drainage method. Figure 5 shows the curves of relative density and relative density of composites with sintering temperature. It can be seen that between 600 °C and 900 °C, the relative density of the composite increases first and then decreases. Compared with 600 °C, when the sintering temperature is 700 °C, the relative density of the composite is greatly improved, reaching 92.8%. This is due to the increase of temperature, which makes the migration rate of Cu grain boundary increase, so that the gap between Cu and CNTs gradually decreases, thus reducing the porosity of the composite and improving its relative density, which is consistent with the microstructure of the composite at this temperature. With the further increase of temperature, the relative density of the composites decreased.

**Figure 6:**
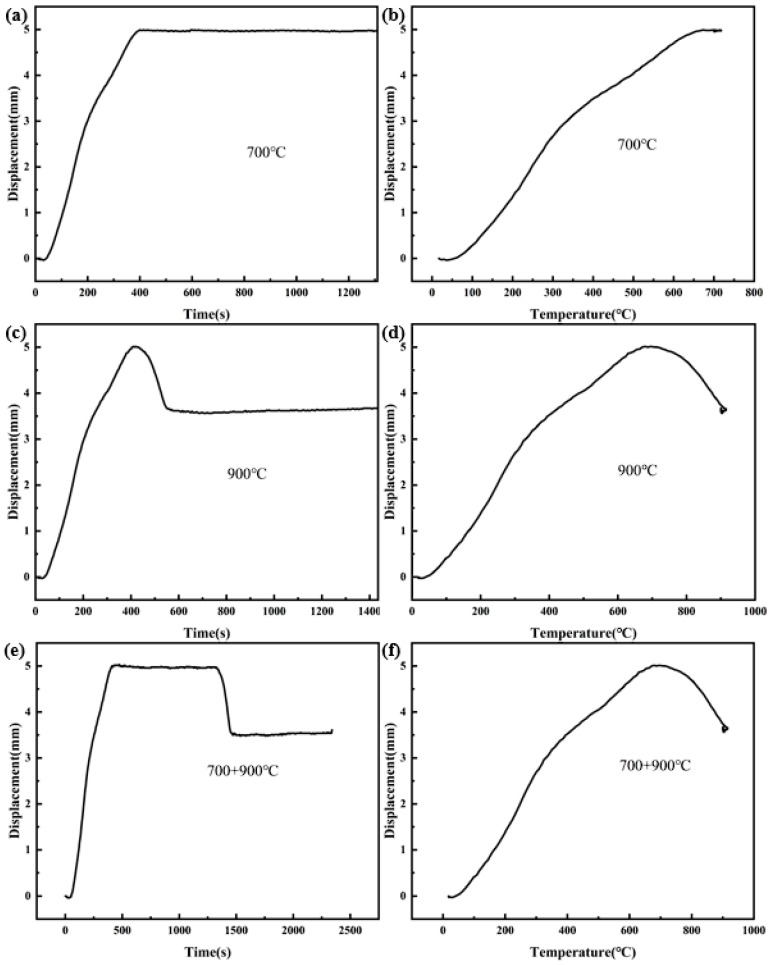
SPS sintering curves at different temperatures. (**a**,**c**,**e**) Displacement-time curve; (**b**,**d**,**f**) Displacement-temperature curve.

The displacement (displacement of the indenter under pressure) during the SPS sintering process can reflect the changing characteristics of the densification of the composite. According to the real-time displacement and temperature in the SPS sintering process, the sintering curve shown in Figure 6 is drawn. When the sintering temperature is lower than 700 °C, the displacement increases with the increase of temperature during the whole sintering process. When the temperature rises to 700 °C, the sintering enters the holding stage. At this time, the displacement remains basically unchanged with the extension of the holding time. When the sintering temperature is greater than 700 °C, the displacement decreases with the increase of temperature. The inflection point temperature in the Figure 6e,f is near 700 °C, and when the temperature rises to 900 °C, the sintering enters the holding stage. At this time, the displacement remains unchanged with the extension of the holding time. It can be seen that the holding time basically has no effect on the relative density of CNTs/Cu composites, and the densification stage of the material is mainly concentrated in the rapid heating stage. In addition, in the rising stage of curve displacement, the change rate of displacement is the largest in the stage of 100~300 °C. The stage is mainly due to the discharge of gas between powders under the action of pressure, which makes the displacement rise, the rising rate is fast and the composite gets denser quickly. The change rate of the curve displacement between 300 °C and 700 °C is small. This stage is mainly under the action of Joule heat and pressure, and further densification under the dual action of plastic deformation and grain boundary diffusion.

**Figure 7:**
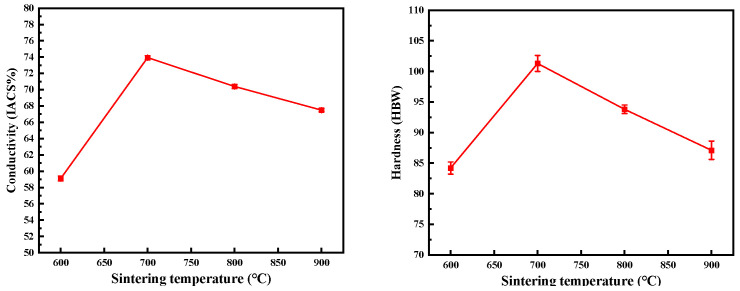
The hardness and electrical conductivity of CNTs/Cu composites with different sintering temperature.

The hardness and electrical conductivity of CNTs/Cu composites prepared at different sintering temperatures are shown in Table 3. Figure 7 shows the curves of hardness and electrical conductivity with the sintering temperature. With the increase of temperature, the hardness and electrical conductivity of the composites increase first and then decrease first, and the relative density shows the same trend. In addition, the higher the sintering temperature, the larger the grain size. According to the Hall-Petch formula, the larger the grain size of the composite, the lower the strength and the lower the hardness. Therefore, the decrease of the hardness of the composite after 700 °C is also positively correlated with the growth of the grain. The conductivity also decreases with the decrease of relative density. Unlike hardness, the larger the grain size, the less the grain boundaries contained in the composite, and the higher the conductivity. Therefore, when the temperature rises after 700 °C, the relative density of the material decreases, which makes the conductivity of the material decrease, while the grain growth makes the conductivity increase, and the relative density plays a leading role.

### 3.3. Directional Rolling

**Figure 8:**
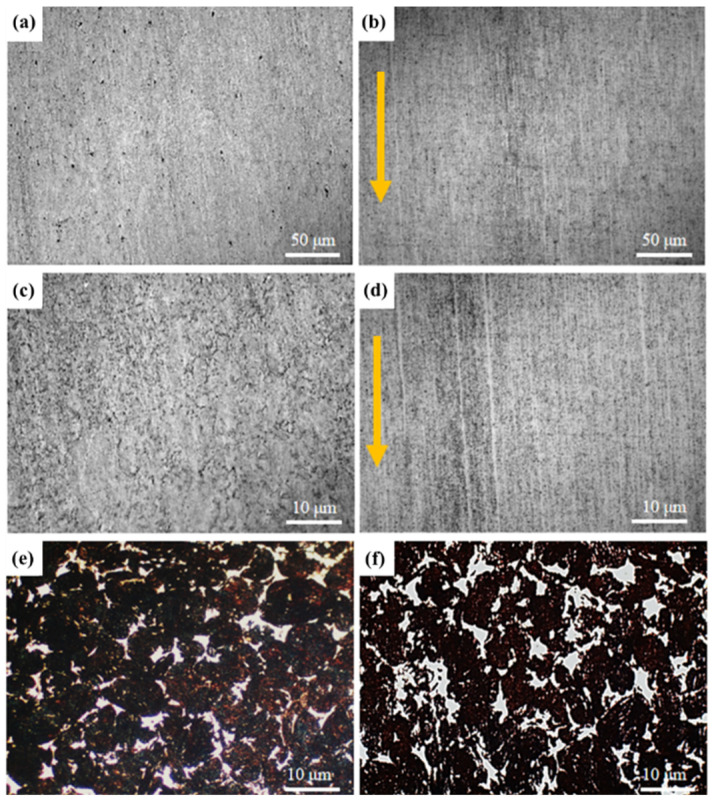
Metallographic microstructure of CNTs/Cu composites before and after rolling. (**a**,**b**) The change of material holes before and after rolling; (**c**,**d**) The change of CNTs before and after rolling; (**e**,**f**) The change of Cu microstructure before and after rolling.

Rolling is an effective method to improve the relative density of composites, which can improve the dispersion and orientation of CNTs, so as to significantly improve the hardness and conductivity of CNTs/Cu composites. In this experiment, the rolling sample (the yellow arrow in the figure is the rolling direction) with the best rolling deformation (60%) was selected (cracks appeared on the surface of CNTs/Cu composites under higher rolling deformation). The microstructure of CNTs/Cu composites before and after rolling was characterized, and the changes of the orientation of holes, CNTs and Cu in the composites before and after rolling were observed, as shown in Figure 8. Before rolling, due to the high content of CNTs in the composite, there is a secondary agglomeration of CNTs during the sintering process, which makes the relative density of CNTs/Cu composite lower. As shown in Figure 8a,c, there are many holes on the surface of the composite. After rolling, under the action of large deformation, the original large holes are squeezed and bridged into small holes or disappear, as shown in Figure 8b,d. At this time, the holes on the surface of the composite are greatly reduced. Figure 8e,f characterizes the change of Cu matrix in the composite. At this time, the metallographic phase of the composite is corroded by the supersaturated FeCl_3_ solution. It can be clearly seen that the Cu particles are elongated along the rolling direction after rolling, and the original oval Cu particles gradually become long strips. Directional rolling greatly improves the dispersion and orientation of CNTs, which is due to the deformation of the original Cu particles under the action of large deformation. The CNTs agglomerated between the Cu particles is also squeezed and sheared by the adjacent particles so that it is dispersed and filled between the Cu particles, thereby improving the relative density of the composite while also making the CNTs more evenly dispersed. Since the controlled rolling direction remains unchanged, the CNTs also has a certain orientation arrangement along the rolling direction. Since the CNTs itself is a one-dimensional tubular structure, its radial and transverse conductivity can be more than 100 times different [23]. This directional arrangement is expected to improve the hardness and electrical conductivity of CNTs/Cu composites at the same time.

**Figure 9:**
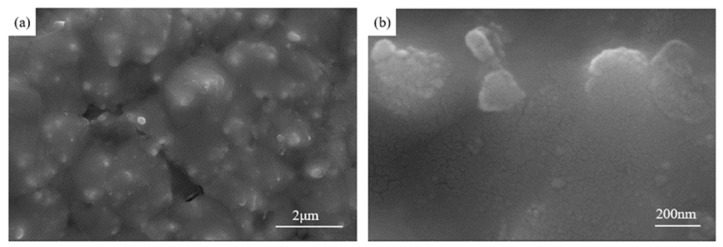
SEM photos of the cross section of the composite after rolling. (**a**) A low magnification; (**b**) High magnification.

In order to further illustrate the orientation of CNTs in the composites after rolling, the cross section perpendicular to the rolling direction of the composites was corroded and the surface morphology was observed, as shown in Figure 9. The area that is significantly different from the contrast of the Cu matrix in the low-magnification image is CNTs, and the obvious CNTs tubular structure can be observed from the high-magnification image. The port of CNTs can be observed at the cross section, which indicates that the CNTs in the matrix is generally parallel to the rolling direction after rolling, and it can be seen that the CNTs are dispersed more evenly, and no obvious agglomeration is observed. This further shows that directional rolling can improve the orientation and dispersion of CNTs, so that CNTs are dispersed twice along the rolling direction.

**Figure 10:**
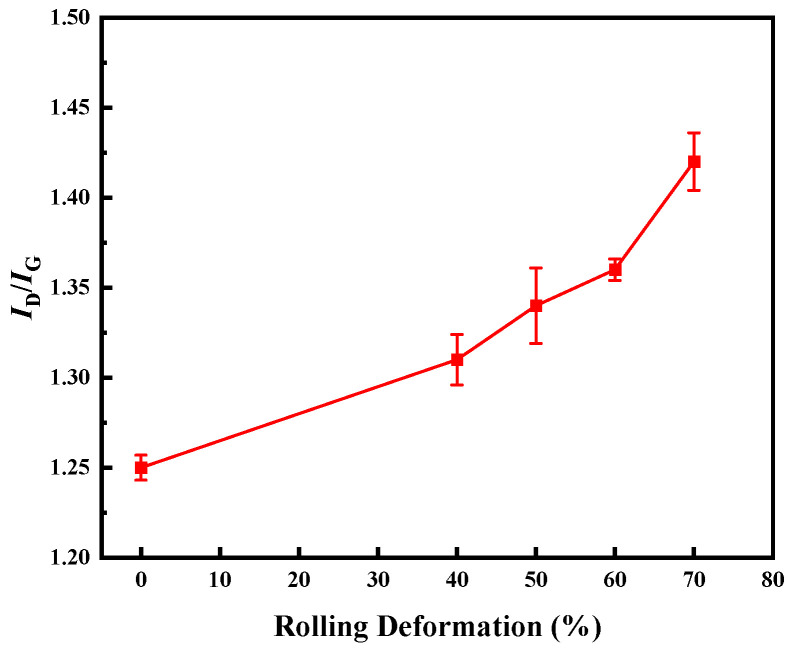
The effect of rolling reduction on the structural integrity of CNTs.

Figure 10 is the change diagram of I_D_/I_G_, reflecting the effect of rolling reduction on the structural integrity of CNTs. The I_D_/I_G_ = 1.25 of CNTs in the composites without rolling; when the rolling deformation is 40%, I_D_/I_G_ = 1.31; when the rolling deformation is 50%, I_D_/I_G_ = 1.34; when the rolling deformation is 60%, I_D_/I_G_ = 1.36; when the rolling deformation is 70%, I_D_/I_G_ = 1.42. With the increase of rolling deformation, the value of I_D_/I_G_ also increases gradually, which indicates that the defects of CNTs increase gradually during the rolling process. This is because the rolling makes the agglomerated CNTs disperse again, which reduces the structural integrity of CNTs. Overall, in the early stage of rolling (0~40%), the value of I_D_/I_G_ increased slowly, and the damage caused by rolling to CNTs was small. With the further increase of rolling deformation, the increase rate of the I_D_/I_G_ value gradually increases. This is because when the rolling deformation is large, the internal stress of the composite is gradually concentrated, and the CNTs at the stress concentration is more vulnerable to damage. When the rolling deformation reaches 70%, cracks appear on the surface of the material, and the stress concentration is large. Compared with the rolling deformation of 60%, the CNTs’ defects increase significantly. On the one hand, increasing the rolling deformation is beneficial to improve the relative density of the composite, thereby improving the conductivity of the composite; on the other hand, the greater the rolling deformation, the more damage to the CNTs, which will affect the conductivity of the CNTs, thereby reducing the conductivity of the CNTs/Cu composite.

**Figure 11:**
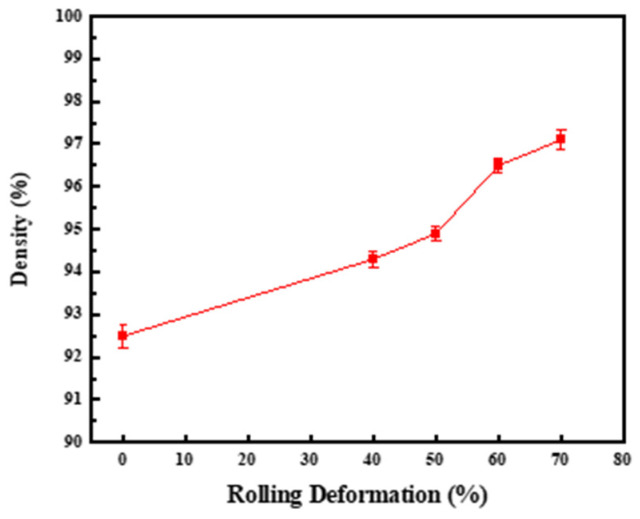
The relative density of 2 wt.% CNTs/Cu composites under different rolling deformations.

Figure 11 is the relative density curve of 2 wt.% CNTs/Cu composites under different rolling deformation. On the whole, with the increase of rolling deformation, the relative density of the composites increases gradually. When the rolling deformation is 40%, the density of the composite increases from 7.68 g/cm^3^ to 7.83 g/cm^3^, and the relative density increases from 92.5% to 94.3%. When the deformation is 40~70%, the increase rate relative density of the composite increases first and then decreases, and the fastest increase is between 50~60%. The relative density of the composite before rolling is only 92.5%, and the relative density of the composite after rolling has reached 97.1%, indicating that the CNTs in the composite after rolling deformation is more evenly distributed in the Cu matrix, so that the pores caused by the secondary agglomeration of CNTs caused by sintering in the composite after rolling are reduced, making the composite more dense. In addition, when the rolling deformation is 70%, the surface of the rolling sample is cracked, and the rolling sample is broken by increasing the reduction.

**Figure 12:**
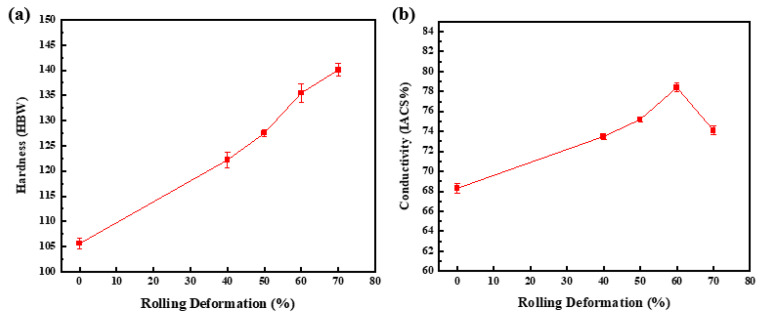
The hardness and electrical conductivity of CNTs/Cu composites with different rolling deformation.

The variation of hardness of 2 wt.% CNTs/Cu composites with rolling deformation is shown in Figure 12a. It can be seen that with the progress of rolling, the hardness of the composite is greatly improved. The greater the rolling deformation, the greater the hardness of the composite, and the relative density of the composite showed the same trend. When not rolled, the porosity of the composite is large, and the CNTs will reunite at the grain boundary during the sintering process, which makes too many CNTs accumulate at the grain boundary of the Cu particles, reducing the bonding force between the grains, resulting in a lower hardness of the composite. Under the action of large deformation, the dispersion of CNTs is more uniform and the bonding with Cu matrix is more closely. When an external force is applied to the composite, due to the close combination between Cu and CNTs, the external load is transferred to the CNTs with high strength and hardness, thereby improving the deformation resistance of the composite and improving the hardness. In addition, rolling can refine the grains, and fine grain strengthening increases the hardness of the composite. When the rolling deformation is 70%, the hardness of the composite increases from 105.6 HBW to 140.1 HBW, which is 34.5% higher than that of the unrolled composite.

The variation of electrical conductivity of 2 wt.% CNTs/Cu composites with rolling deformation is shown in Figure 12b. The conductivity of the composites increases first and then decreases with the increase of rolling deformation and does not show the same single upward trend as the relative density and hardness. This is because the conductivity of the CNTs/Cu composites is affected by many factors. It is generally believed that the relative density of the material, the interface area and the degree of dispersion and integrity of CNTs are the main factors affecting the conductivity of CNTs/Cu composites.

When the rolling deformation is between 0 and 60%, the conductivity of the composite increases from 68.3%IACS to 78.4%IACS, an increase of 14.8%. This is mainly because the increase of the amount of deformation makes the relative density of the composite increase and the porosity decrease. The pores will affect the movement of free electrons. In addition, the dispersion degree of CNTs is gradually increased, which makes the conductivity of the composite increase. The rolling deformation decreased from 60% to 70%, and the conductivity decreased from 78.4%IACS to 74.1%IACS, which was caused by many factors. On the one hand, with the increase of rolling deformation, the lattice integrity of CNTs is gradually destroyed while CNTs is gradually dispersed, and the generation of defects will reduce the conductivity of CNTs, thus reducing the overall conductivity of CNTs/Cu composites. On the other hand, rolling will refine the grain size of the composite, thereby increasing the grain boundary area, and the grain boundary will hinder the movement of electrons, thereby reducing the conductivity of the composite. In general, in the 0~60% stage, the increase of relative density and CNTs dispersion plays a leading role, which makes the conductivity of the composite increase. In the 60~70% stage, the increase of grain boundary area and the increase of CNTs damage degree make the conductivity of the composites decrease.

Table 4 summarizes the properties of CNTs/Cu composites in some literature. Overall, the hardness of the CNTs/Cu composite prepared in this study reached a high level, mainly due to the optimization of the preparation process in this paper, which achieved uniform dispersion of high content CNTs. The hardness has been improved through rolling deformation process, and the conductivity is relatively high, indicating that the structural damage to CNTs is relatively small. Finally, CNTs/Cu composites have good hardness and conductivity.

## 4. Conclusions

In this paper, the densification sintering process of CNTs/Cu composites with a high CNTs content was explored. The relative density of the composites was further improved and the directional arrangement of CNTs was realized by directional rolling. The effect of CNTs content on the microstructure and properties of the composites was studied. Finally, CNTs/Cu composites with high hardness and high conductivity were prepared. The main conclusions are as follows:

(1) The composites were prepared by vacuum hot pressing sintering and SPS sintering. The CNTs/Cu composites prepared by SPS had higher relative density and better overall performance than HPS.

(2) For SPS sintering, if the sintering temperature is too low, it is difficult to sinter and densify. If the sintering temperature is too high, CNTs will agglomerate again to reduce the relative density of the material. With the increase of sintering temperature, the relative density of high content CNTs/Cu composites prepared by SPS increased first and then decreased and the relative density was the highest at 700 °C, reaching 92.8% (1.5 wt.%).

(3) Directional rolling reduced the porosity of CNTs/Cu composites (2 wt.%), increased the relative density and improved the dispersion and orientation of CNTs. With the increase of rolling deformation (40%, 50%, 60%, 70%), the hardness of the composites increased gradually, and the conductivity increased first and then decreased. When the rolling deformation is 60%, the composite has the best comprehensive performance. Compared with that before rolling, the hardness increased by 28.6% and the conductivity increased by 14.8%.

In the future, we will continue to study the plasticity, wear resistance and high temperature resistance of CNTs/Cu composites.

## Figures and Tables

**Table 1:** Statistical table of material relative density and relative density under different sintering methods.

Serial Number	The Mode of Sintering	Actual Density (g/cm^3^)	Relative Density (%)	Hardness (HBW)	Electric Conductivity (%IACS)
1-1	HPS, 1 wt.% CNTs	7.41 ± 0.22	86.1 ± 1.8	72.0 ± 0.2	68.0 ± 1.3
1-2	HPS, 2 wt.% CNTs	7.07 ± 0.30	85.2 ± 2.5	82.6 ± 0.5	55.4 ± 1.5
1-3	SPS, 1 wt.% CNTs	7.92 ± 0.17	92.0 ± 1.5	84.3 ± 0.5	79.4 ± 0.8
1-4	SPS, 2 wt.% CNTs	7.56 ± 0.26	91.1 ± 2.2	96.2 ± 0.6	66.5 ± 0.8

**Table 2:** Statistics of density and relative density of materials at different sintering temperatures.

Serial Number	Sintering Temperature (°C)	Actual Density (g/cm^3^)	Relative Density (%)
2-1	600	6.95 ± 0.19	82.3
2-2	700	7.84 ± 0.13	92.8
2-3	800	7.64 ± 0.24	90.4
2-4	900	7.49 ± 0.17	88.6
2-5	1000	/	/

**Table 3:** Statistics of hardness and conductivity of materials at different sintering temperatures.

Serial Number	Sintering Temperature (°C)	Hardness (HBW)	Electric Conductivity (%IACS)
2-1	600	84.2 ± 0.7	59.1 ± 1.3
2-2	700	101.3 ± 1.2	73.9 ± 0.6
2-3	800	93.8 ± 1.0	70.4 ± 0.8
2-4	900	87.1 ± 0.5	67.5 ± 0.5

**Table 4:** Performance comparison of reference materials.

Reference	Composite	Hardness	Electric Conductivity
Shaoli Fu [20]	0.5 vol.% CNTs/Cu	102.5 HV	92.9%IACS
Xiuhuo Guo [26]	1.45 wt.%CNTs/Cu	39.9 HBW	66.8%IACS
	0.35 wt.%CNTs/TiB_2_/Cu	58.8 HBW	56.9%IACS
Zixin Huang [27]	1.25 wt.%CNTs/Cu	110.1 HV	-
Bohua Duan [28]	0.5 wt.%CNTs/Cu	84 HV	-
Yu Pan [29]	1.5% wt.%CNTs/Cu	97 HV	89.6%IACS
	1.5 wt.%CNTs/0.5 wt.%Al_2_O_3_/Cu	131 HV	87.9%IACS
A.K.Shukla [18]	2.5 wt.%MWCNT/Cu	53 HBW	-
	2.5 wt.%SWCNT/Cu	105 HBW	
Siwei Luo [25]	CNTs/Cu	110.6 HV	-
	CNTs@Cr_2_O_3_/Cu	117.3 HV	
This Work	2 wt.%CNTs/Cu	140.1 HBW	78.4%IACS

## Data Availability

The original contributions presented in the study are included in the article, further inquiries can be directed to the corresponding authors.
